# Full-Length Genome Sequence of a Sindbis Virus Strain Isolated from Culex cinereus in 1977 in Bozo, Central African Republic

**DOI:** 10.1128/genomeA.00455-18

**Published:** 2018-06-28

**Authors:** Rita C. Sem Ouilibona, Huguette D. Tchetgna Simo, Ulrich Vickos, Nicolas Berthet, Emmanuel Nakouné

**Affiliations:** a Institut Pasteur de Bangui, Bangui, Central African Republic; b Centre Interdisciplinaire de Recherches Médicales de Franceville (CIRMF), Franceville, Gabon; c Institut Pasteur, Unité Environnement et Risques Infectieux, Cellule d’Intervention Biologique d’Urgence, Paris, France; d Centre National de Recherche Scientifique (CNRS) UMR3569, Paris, France

## Abstract

We report here the complete genome sequence of a Sindbis virus (SINV) strain, ArB7761, isolated in 1977 in the Central African Republic. This strain, closely related to the Babanki virus, belongs to the SINV genotype I clade. However, it differs from the Egyptian prototype strain AR339 by several indels in the nsP3 gene.

## Abstract (precis)

We report here the complete genome sequence of a Sindbis virus (SINV) strain, ArB7761, isolated in 1977 in the Central African Republic. This strain, closely related to the Babanki virus, belongs to the SINV genotype I clade.

## GENOME ANNOUNCEMENT

The Sindbis virus (SINV) is the prototype of the arthropod-borne virus (arbovirus) genus Alphavirus, a member of the Togaviridae family. In humans, SINV infections are mainly benign, consisting of fever, rash, arthralgia, and myalgia (1), but no virus-specific vaccines or therapies for the treatment of this disease are currently available (2). Children are often infected but develop only subclinical disease, while clinical symptoms are more prevalent in adults. Morbidity is highest in women between 45 and 65 years of age (3). This zoonotic virus is maintained in nature through a transmission cycle involving birds and Culex mosquitos. Since its initial description in Egypt in 1952, SINV has been found in Africa, Asia, Australia, and Europe (Scandinavia and Russia) (4), although clinical infections and outbreaks have been described in South Africa and northern Europe only. Currently, six geographically distinct genotypes of SINV are recognized, but the virus is sometimes referred to as Babanki, Ockelbo, Karelian fever, Kyzylagach, or Whataroa, depending on location.

SINV strain ArB7761 was isolated on suckling mice from a pool of Culex cinereus mosquitoes collected in a forested area in 1977 at Bozo (05°18ʹN, 18°28ʹE), Central African Republic (CAR). The lyophilized virus was resuspended in phosphate-buffered saline and amplified on Vero E6 cell lines. Cytopathic effects were observed 2 to 3 days postinfection, and then the harvested supernatant was used for RNA extraction using the QIAamp viral RNA minikit (Qiagen). Double-stranded cDNA was synthetized and used for paired-end library construction with the NEBNext Ultra RNA library prep kit for Illumina (New England BioLabs, UK). High-throughput sequencing was performed on a MiSeq instrument (Illumina). A total of 4,685,922 paired-end reads were obtained, and 195,973 reads corresponded to SINV. Analysis revealed a sequence of 11,709 bp in length. Our sequence shows 99% similarity with the Babanki virus (GenBank accession number HM147984) from Cameroon and 94% similarity with the AR339 prototype SINV strain (J02363). At the amino acid level, similar to most alphaviruses, there is an additional stop codon between genes for nonstructural proteins nsP3 and nsP4. Compared with AR339, five amino acids (codons) (M, Q, E, A, and D) are inserted at positions 1786, 1787, 1788, 1799, and 1800 of the nonstructural polyprotein nsP123, respectively, whereas three amino acids (codons) (A, V, and Q) are deleted at positions 1827, 1828, and 1829 at the end of nsP3, respectively. These mutations been already demonstrated in other SINV strains (5). Furthermore, 22 and 57 amino acid changes were observed in the structural and nonstructural polyproteins, respectively. A maximum likelihood phylogenetic tree shows that SINV_CAR_1976 belongs to genotype I of SINV, along with the other African strains (6).

In conclusion, these molecular data on our new SINV isolate support the wide distribution of this virus in Africa. However, given our limited knowledge of this virus and the reasons why cases remain limited to a few regions, further studies are required to better understand its transmission cycles.

### Accession number(s).

The complete genome sequence of SINV strain ArB7761 was deposited in GenBank under the accession number MH212167.

## References

[1] Suhrbier A, Jaffar-Bandjee M-C, Gasque P 2012 Arthritogenic alphaviruses—an overview. Nat Rev Rheumatol 8:420–429. doi:10.1038/nrrheum.2012.64. 22565316

[2] Russell DL, Dalrymple JM, Johnston RE 1989 Sindbis virus mutations which coordinately affect glycoprotein processing, penetration, and virulence in mice. J Virol 63:1619–1629. 2926866 10.1128/jvi.63.4.1619-1629.1989 PMC248406

[3] Adouchief S, Smura T, Sane J, Vapalahti O, Kurkela S 2016 Sindbis virus as a human pathogen—epidemiology, clinical picture and pathogenesis. Rev Med Virol 26:221–241. doi:10.1002/rmv.1876. 26990827

[4] Kurkela S, Manni T, Myllynen J, Vaheri A, Vapalahti O 2005 Clinical and laboratory manifestations of Sindbis virus infection: prospective study, Finland, 2002–2003. J Infect Dis 191:1820–1829. doi:10.1086/430007. 15871114

[5] Simpson DA, Davis NL, Lin S-C, Russell D, Johnston RE 1996 Complete nucleotide sequence and full-length cDNA clone of S.A.AR86, a South African alphavirus related to Sindbis. Virology 222:464–469. doi:10.1006/viro.1996.0445. 8806532

[6] Lundström JO, Pfeffer M 2010 Phylogeographic structure and evolutionary history of Sindbis virus. Vector Borne Zoonotic Dis 10:889–907. doi:10.1089/vbz.2009.0069. 20420530

